# Update on the implication of potassium channels in autism: K^+^ channelautism spectrum disorder

**DOI:** 10.3389/fncel.2015.00034

**Published:** 2015-03-02

**Authors:** Luca Guglielmi, Ilenio Servettini, Martino Caramia, Luigi Catacuzzeno, Fabio Franciolini, Maria Cristina D’Adamo, Mauro Pessia

**Affiliations:** ^1^Section of Physiology and Biochemistry, Department of Experimental Medicine, University of Perugia School of Medicine, PerugiaItaly; ^2^Department of Chemistry, Biology and Biotechnology, University of Perugia, PerugiaItaly

**Keywords:** K^+^ channels, Kv4.2 (*KCND2*), Kv7.3(*KCNQ3*), K_Ca1.1_ (*KCNMA1*), Kir2.1 (*KCNJ2*), Kir4.1 (*KCNJ10*), FMRP (*FMR1*), channelASD-channelepsy phenotype

## Abstract

Autism spectrum disorders (ASDs) are characterized by impaired ability to properly implement environmental stimuli that are essential to achieve a state of social and cultural exchange. Indeed, the main features of ASD are impairments of interpersonal relationships, verbal and non-verbal communication and restricted and repetitive behaviors. These aspects are often accompanied by several comorbidities such as motor delay, praxis impairment, gait abnormalities, insomnia, and above all epilepsy. Genetic analyses of autistic individuals uncovered deleterious mutations in several K^+^ channel types strengthening the notion that their intrinsic dysfunction may play a central etiologic role in ASD. However, indirect implication of K^+^ channels in ASD has been also reported. For instance, loss of *fragile X mental retardation protein* (FMRP) results in K^+^ channels deregulation, network dysfunction and ASD-like cognitive and behavioral symptoms. This review provides an update on direct and indirect implications of K^+^ channels in ASDs. Owing to a mounting body of evidence associating a channelopathy pathogenesis to autism and showing that nearly 500 ion channel proteins are encoded by the human genome, we propose to classify ASDs - whose susceptibility is significantly enhanced by ion channels defects, either in a monogenic or multigenic condition - in a new category named “***c****hannel****A****utism* ***S****pectrum* ***D****isorder*” (*channelASD; cASD*) and introduce a new taxonomy (e.g., K_v_*x.y*-*channelASD and likewise* Na_v_*x.y*-*channelASD*, Ca_v_*x.y*-*channelASD; etc.*). This review also highlights some degree of clinical and genetic overlap between K^+^ channelASDs and K^+^ channelepsies, whereby such correlation suggests that a subcategory characterized by a *channelASD-channelepsy phenotype* may be distinguished. Ultimately, this overview aims to further understand the different clinical subgroups and help parse out the distinct biological basis of autism that are essential to establish patient-tailored treatments.

## INTRODUCTION

Nearly 70 million people worldwide suffer from ASD. 700,000 are from USA and the estimated cost to society of caring for these children has been $11.5 billion in 2011 (Lavelle et al., 2014). ASD is a group of heterogeneous neurodevelopmental disorders previously dealt with as single pathologies: autistic disorder, pervasive developmental disorder-not otherwise specified (PDD-NOS), Asperger syndrome, childhood disintegrative disorder also known as Heller syndrome, and Rett syndrome. Specific impairments before age three, in each of the following three core domains fit the diagnostic criteria for ASD: ***social*** (difficulty in interacting with people, in reading facial expressions and making eye contact), ***language*** (difficulty in using or understanding language, tending to focus attention and conversation on a limited number of topics, frequently repeat phrases, and have very limited speech ability), and ***restricted/repetitive behavior*** (excessive attention to routines and difficulty in adjusting to new surroundings or changes in routine). Given the complexity of the disorder and the variety and severity of the symptoms, ASD is thought to be caused by multiple factors interacting in a complex way. Genetic defects, both inherited or occurring spontaneously, undoubtedly play a role in increasing the susceptibility to the disorder by affecting brain development and proper neural networking. Neurological comorbidities are often observed in ASD patients and they are associated with more clinical severity. The most noted is epilepsy, comorbid in 30% of ASD, about 50 times higher than in the general population (Tuchman and Rapin, 2002; Mouridsen et al., 2011). Cognitive and behavioral deficits occurs in up to 46% of children with epilepsy (Clarke et al., 2005; Matsuo et al., 2010) suggesting that, at least in these cases, the excitability imbalance could be a common denominator. Another comorbidity observed in nearly 90% of genetic syndromes associated with ASDs is motor delay. Delays occur in both gross and fine motor domains, while deficits are documented in praxis, motor planning, gait, coordination, and postural control (Rinehart and McGinley, 2010; Van Waelvelde et al., 2010). Several lines of evidence suggest that motor delay is caused by aberrant neural circuitry mostly in the cerebellum and fronto-striatal regions (Rinehart et al., 2006; Wegiel et al., 2013).

The relative risk for a child to develop the disease is increased ~25-fold in families in which a sibling is affected (Jorde et al., 1991). Unfortunately, the genetic etiology of autism is characterized by high locus heterogeneity, where *de novo* and rare inherited CNVs and SNV mutations in conjunction contribute to the overall genetic risk to develop the disease. To date, we know that defined mutations, genetic syndromes and *de novo* CNVs account for about 30% of ASD cases (Schaaf and Zoghbi, 2011). Linkage analyses of ASD patients have allowed the identification of *loss-* or *gain-of-function* mutations and chromosomal alterations in genes coding for several channel types, including K^+^ channels. These proteins are highly heterogenic and widely expressed in the CNS where they set the resting membrane potential of neurons and glia, shape action potentials and regulate firing, neurotransmitter release and wiring. At network level, K^+^ channels are involved in post-synaptic EPSP-spike coupling, activity dependent short- and long-term synaptic plasticity and information processing. Thus, these channels provide neurons, glia and CNS networks with distinct electrical identity and their dysfunction is a major contributing factor in excitability disequilibrium and network impairment. Animal models, in which these genes are deleted or mutated, often show epilepsy (Eijkelkamp et al., 2012; D’Adamo et al., 2013, 2014), motor impairment, learning and behavioral phenotypes that resemble somehow ASD. The insights, provided by investigations with these animal models, are valuable to understand neural networks abnormalities potentially underlying ASD. The plethora of ion channel types expressed by neurons and the numerous variations discovered in their relevant genes often generate confusion in the classification of the associated diseases. To further understand the clinical subgroups of autism, help parse out distinct biological basis of autism and identify tailored treatments, we propose to name the ion channels defects contributing significantly to either monogenic or multigenic ASDs “*channelASD*” and suggest a new taxonomy (e.g., K_v_*x.y*-*channelASD and likewise* Na_v_*x.y*-*channelASD*; Ca_v_*x.y*-*channelASD; etc.*), according to a previously proposed nomenclature for ion channels dysfunctions underlying epilepsy (D’Adamo et al., 2013). Interestingly, this review will also show that there is a high degree of clinical overlap between *channelASD* and *channelepsy*. This strong correlation between a distinct subgroup of patients prompt us to also define a new phenotype: “*channelASD-channelepsy,”* according with our previously and presently introduced nomenclature.

## VOLTAGE-GATED K^+^ CHANNELS

The first voltage-gated K^+^ channel (Kv) was cloned from the *Shaker* mutant of *Drosophila melanogaster* (Tempel et al., 1987). Since then, a number of other genes encoding for Kv channels have been identified from many different species. Based on sequence relatedness, Kv channels have been classified in subfamilies from Kv1 to Kv12 (Chandy and Gutman, 1993). The α subunit contains six transmembrane segments with the *N*- and *C*-termini residing inside the cell. The full crystal structure, provided for a Kv channel, confirmed that this channel is composed of four homologous pore-forming α subunits. These channels may exist either as homomers, whenever four identical α-subunits are assembled or as heteromers, whenever different types of α-subunits heteropolymerize to form channels with distinct functional and pharmacological properties (Pessia, 2004).

### K**v**4.2 – ChannelASD

*KCND2* gene encodes for the α subunit of the voltage-gated K^+^ channel Kv4.2 that generates an “A-type” current (*I_A_*). When assembled in homotetramers, Kv4.2 channels activate at sub-threshold membrane potentials, inactivate rapidly, and quickly recover from inactivation (Zhu et al., 1999; Birnbaum et al., 2004). The availability of this channel type, in response to membrane potential fluctuation, is regulated by its peculiar inactivation kinetics occurring from the open state by a fast *N*-type mechanism or (*much more frequently*) from pre-open closed states, when the voltage-sensor domains activate but the pore fails to open (Bahring et al., 2001; Gebauer et al., 2004; Jerng et al., 2004; Barghaan and Bahring, 2009; Bahring and Covarrubias, 2011). *In vivo*, other proteins further tune-up Kv4.2 inactivation kinetics: the K^+^ channel interacting protein (KChIP) and the dipeptidyl-peptidase-like protein (DPP) that respectively inhibits and accentuates N-type open state inactivation (Wang et al., 2002; Jerng et al., 2005, 2009; Covarrubias et al., 2008). This channel type is mainly expressed in the hippocampal CA1 pyramidal neurons where it regulates the threshold for action potential initiation and repolarization, frequency-dependent AP broadening, and back-propagation of action potentials (Chen et al., 2006; Kim et al., 2007; Nerbonne et al., 2008). These notions were further corroborated by the evidence that Kv4.2 channel deletion lowers and increases the threshold for LTP and LTD induction, respectively (Chen et al., 2006; Zhao et al., 2011). Conversely, enhancement of Kv4.2 expression prevents LTP induction (Jung et al., 2008). The presence of Kv4.2 channels in hippocampus appears fundamental, mostly at early developmental stage when neuronal activity drives synaptic maturation and network refinement. At hippocampal synapses, the gradual reduction in GluN2B/GluN2A subunit ratio, during post-natal development, is correlated with AMPA expression and synaptic maturation. Ablation of Kv4.2 in mice abolished this phenomenon and resulted in a higher number of silent synapses in the adulthood (Kim and Hoffman, 2012). Noteworthy, both NMDA subunit composition and synaptic inactivity are rescued by reentering the channel (Kim and Hoffman, 2012).

Given the importance of Kv4.2 in brain development and functioning, defects of this channel have been unsurprisingly correlated with a broad spectrum of neurological disorders. Gene deletion in mice leads to increased susceptibility to convulsant stimuli (Barnwell et al., 2009) and truncating mutation of Kv4.2 in humans leads to temporal lobe epilepsy (Singh et al., 2006). Moreover, rare variants in *KCND2* have been identified in individuals with autism, namely: a submicroscopic *de novo* deletion (Okamoto et al., 2011), three translocation breakpoints (at 7q22.1, 7q31.2 and 7q31.3) potentially deleterious (Scherer et al., 2003), and three substitution variants, (N544S; F538S; R539L) reported in three independent cases of autism (Mikhailov et al., 2008). Recently, exome sequencing of two identical twins affected by autism, with frequent and uncontrollable seizures, revealed a *de novo* heterozygous variant in *KCND2* gene that resulted in the substitution V404M (Lee et al., 2014). Expression and functional characterization of Kv4.2 V404M channels in *Xenopus laevis* oocytes showed significantly slower inactivation than wild-type, leading to grater channel availability in response to depolarization. These functional alterations persisted when the mutated subunit was co-expressed with the wild-type (in 1:1 ratio to mimic the heterozygous nature of the variation), suggesting a dominant effect. The V404M impact on channel kinetics was attributed to impaired closed-state inactivation, because the effect was still evident in the presence of the auxiliary subunits KChIP3a or DPP10a (Lee et al., 2014).

### K**v**4.2 – ChannelASD linked to Fragile X syndrome

Kv4.2 channel expression may also participate in establishing the conditions for the development of ASDs, given that Kv4.2 mRNA can bind to the *fragile X mental retardation protein* (FMRP), which is associated to *fragile X syndrome* (FXS), the most common monogenic cause of autism and inherited intellectual retardation (Garber et al., 2008) often accompanied by seizures and poor motor coordination (Garber et al., 2008). FXS is present in ~5% of ASDs cases, and is the most likely cause of autism in those individuals. FXS is caused by an expansion of CGG triplets (>200) in the 5^′^ untranslated region of the fragile X mental retardation gene 1 (*FMR1*), located on chromosome X (Krawczun et al., 1985; Fu et al., 1991), which results in the failure to produce the FMRP, required for normal neural development (Pieretti et al., 1991; Verheij et al., 1993; Santoro et al., 2012). This protein is predominantly post-synaptic, where is associated with ribosomal complexes and represses or promotes the translation of specific mRNAs (Siomi et al., 1993; Todd and Malter, 2002; Zalfa et al., 2003; Weiler et al., 2004; Muddashetty et al., 2007; Bechara et al., 2009). The function and localization of this protein make it a key regulator of synaptic plasticity, especially for all those activity dependent processes that require the synthesis of new proteins. In particular, FRMP appears to play a critical role in the maintenance of LTD and LTP in the hippocampus (Weiler et al., 1997; Huber et al., 2000, 2002; Hou et al., 2006; Lauterborn et al., 2007; Michalon et al., 2012). In hippocampal dendrites, FMRP is associated with Kv4.2 mRNA and prevents its translation leading to reduced channel expression at post-synaptic membrane (Lee et al., 2011). Consistent with this scenario, hippocampal neurons from *Fmr1-*null mice show higher levels of Kv4.2 protein than WT and reduced LTP, when induced by threshold stimuli (Lee et al., 2011; **Table 2**). The application of heteropodatoxin (HpTx2), a spider toxin that specifically blocks Kv4.2 channels, restores LTP in hippocampal slices derived from *Fmr1-*null mice (Lee et al., 2011). It is therefore likely that deregulated expression of Kv4.2, resulting from FMRP-dependent abnormalities, may contribute significantly to FXS pathogenesis and generate the conditions for ASDs development.

### K_**v**_7.3 – ChannelASD

The K^+^ channel subunit Kv7.3 is encoded by the *KCNQ3* gene and is expressed in the hippocampus and cortex where it assembles predominantly with the homologous Kv7.2 to form “M” channels (Wang et al., 1998). Generation of functional M channels by heteropolymerization of Kv7.2(*KCNQ2*) with Kv7.4(*KCNQ4*) or Kv7.5(*KCNQ5*) subunits has also been described (Kubisch et al., 1999; Schroeder et al., 2000). This channel type was identified by Brown and Adams (1980) as the principal mediator of muscarine-induced depolarization. They are located in electrically critical regions of neuronal membrane, generate a typical sub-threshold K^+^ conductance and regulate the excitability of many types of neurons. In hippocampal pyramidal cells, the channel is found in the peri-somatic regions where it modulates EPSPs integration and set the threshold for EPSP-spike coupling (Shah et al., 2008). The expression of M channels is finely tuned during the development of early forms of neuronal synchrony. Indeed, in immature CA3 pyramidal cells, the “*giant depolarizing potentials*” (GDPs) exist thanks to the excitatory nature of GABA in synergy with M channel down-regulation. The progressive disappearance of GDPs, following the first postnatal week, is matched by enhancement of M current density (Safiulina et al., 2008) and development of more organized forms of activity, such as theta and gamma rhythms (Buzsáki and Draguhn, 2004).

*KCNQ3* and *KCNQ2* gene mutations segregate with various forms of Kv7.3/Kv7.2-channelepsies (Maljevic et al., 2008; D’Adamo et al., 2013) such as benign familial neonatal convulsion (BFNC) and rolandic epilepsy. Importantly, >20% of patients with rolandic epilepsy have cognitive deficits and >10% display abnormal behavioral (ADHD, anxiety, depression, and pervasive developmental disorder; Tovia et al., 2011). Furthermore, 40% of patients with BFNC show delayed psychomotor development or intellectual disability (Steinlein et al., 2007). Several *KCNQ3* mutations, associated with BFNC, reduce heteromeric M current amplitudes (Singh et al., 2003). Mice carrying the heterozygous G311V mutation in the channel’s pore show a lower threshold for epileptogenesis. While, homozygous mice exhibit spontaneous tonic–clonic seizures associated with reduced M current amplitudes and increased deactivation kinetics in hippocampal CA1 pyramidal cells (Singh et al., 2008). Interestingly, a *de novo* interstitial deletion in the long arm of chromosome 8, encompassing *KCNQ3*, has been identified in two children with a broad spectrum of congenital abnormalities, psychomotor delay and convulsions (Verheij et al., 2009). Another study from Gilling et al. (2013) identified the *de novo* reciprocal translocation *t*(3;8)(q21, q24) truncating *KCNQ3* gene and the missense mutation P574S in distinct autistic patients. The *de novo* translocation was identified in a Danish boy affected by idiopathic periodic trembling since the age of 2 days that persisted for the first 5 weeks of life. In one case, the P574S variant, which substitutes a phylogenetically conserved residue in the *C*-terminal region of Kv7.3, was inherited from the mother who suffered from major depression. Surprisingly, none of the patients harboring this variant had histories of seizures. Functionally, the P574S variant significantly reduces K^+^ current amplitude in oocytes when co-expressed with Kv7.5 subunit but not with Kv7.2 or Kv7.4 subunits (Gilling et al., 2013; **Table 1**). Given the importance of M current in the development of neuronal identity and regulation of excitability, its impairment in immature neurons can depolarize membrane potential increasing cell excitability, facilitating the onset of seizures and delaying the definition of complex neuronal rhythms that possibly result in autistic phenotype.

**Table 1:** Direct involvement of K^+^ channels in ASD.

Gene, channel, neurophysiological role	KO/cKO phenotype	Epilepsy: genetic mutation or chromosomal aberration/phenotype	ASD: genetic mutation or chromosomal aberration/phenotype	Reference
***KCND2/*Kv4.2:** – Neuronal: regulates the threshold for action potential initiation and repolarization, frequency-dependent AP broadening, and back-propagation of AP.	*– Kcnd2*^-/-^: display increased susceptibility to convulsant stimulation.	– Truncating mutation of Kv4.2: temporal lobe epilepsy.	– V404M: *gain-of-function:* slows channel inactivation leading to grater channel availability in response to depolarization. Found in two identical twins affected by autism, with frequent and uncontrollable seizures.	Chen et al. (2006), Singh et al. (2006), Kim et al. (2007), Nerbonne et al. (2008), Barnwell et al. (2009), Lee et al. (2014)

***KCNQ3*/Kv7.3:** – Neuronal: generation of sub-threshold potassium current *I_M_* which, in the hippocampus, modulates EPSPs integration and set the threshold for EPSP-spike coupling.		– G311V pore mutation: benign neonatal epilepsy. – *De novo* interstitial deletion in the long arm of chromosome 8 encompassing *KCNQ3*: psychomotor delay and convulsions.	– *De novo* reciprocal translocation t (3; 8) (q21, q24) truncating *KCNQ3*. Found in one patient with delayed verbal and social development affected by idiopathic periodic trembling. – P574S: *loss-of-function* significantly reduces potassium current amplitude in *Xenopus laevis* oocytes when co-expressed with Kv7.5 subunit but not with Kv7.2 or Kv7.4 subunits. Found in three patients affected by autism.	Singh et al. (2003, 2008), Shah et al. (2008), Verheij et al. (2009), Gilling et al. (2013)

***KCNMA1*/K_**Ca**_1.1** – Neuronal: negatively regulates Ca^2+^ entry and provides a damping mechanism for excitatory signals.	– K_Ca_1.1^-/-^ and PNs-K_Ca_1.1 ^-/-^ : display deficit in cerebellar learning, ataxia, abnormal locomotion and pronounced lack of coordination.	*– KCNMA1 gain-of-function* mutations: cause generalized epilepsy and paroxysmal dyskinesia.	– A138V: the substitution creates a cryptic splice donor site in the second exon. Found in one patient affected by epilepsy, impairments of social interactions and communications skills, lack of spoken language and poor communicating gestures. *– De novo* 9q23/10q22 translocation destructing *KCNMA1*: found in one patient affected by impairments of social interactions and communication skills, lack of spoken language and poor communicating gestures.	Lancaster and Nicoll (1987), Robitaille and Charlton (1992), Shao et al. (1999), Hu et al. (2001), Du et al. (2005), Laumonnier et al. (2006), Womack et al. (2009)

***KCNJ2/*Kir2.1** – Glial: in combination with Kir4.1 controls astrocyte-mediated K^+^ buffering. – Neuronal: plays an important role in DGCs firing properties during development.		– *KCNJ2 loss-of-function* mutation: cause Andersen-Tawil syndrome a condition characterized by long QT-syndrome, cardiac arrhythmia, skeletal abnormalities, mood disorders and seizures.	– K346T: *gain-of-function:* leads to (i) enhanced the channel’s stability at the plasma membrane; (ii) reduced protein ubiquitylation and degradation; (iii) altered protein compartmentalization in lipid rafts, by targeting more channels to cholesterol-poor domains; (iv) reduced interactions with caveolin 2. Found in two identical twins affected by epilepsy, impaired social interaction, absence of speech, repetitive behaviors and intellectual disability.	Bordey and Sontheimer (1998), Haruna et al. (2007), Jabs et al. (2008), Chan et al. (2010), Ambrosini et al. (2014)

***KCNJ10*/Kir4.1** – Glial: potassium buffering, astrocytes and oligodendrocytes differentiation. – Neuronal:(regulates) expressed in *locus coeruleus* neurons	*– Kcnj10^-/-^* : displays severe motor deficits, ipomyelinization in the spinal cord, severe spongiform vacuolation and damaged astrocytes. – cKO *gfa2* directed*:* displays pronounced body tremor, lethargy and ataxia as well as visual placing deficiency and stress induced seizures.	– *KCNJ10 loss-of-function* mutations: cause EAST or SeSAME syndrome.	– R18Q: *gain-of-function*, leads to increased channel surface expression. Found in two identical twins affected by epilepsy, impaired social interaction, absence of speech, repetitive behaviors and intellectual disability. – V84M: *gain-of-function*, leads to increase channel unit conductance compared to WT. Found in one patient affected by poor social gaze, no response to name, absence of language development and seizures.	Orkand et al. (1966), Neusch et al. (2001), Djukic et al. (2007), Higashimori and Sontheimer (2007), Bockenhauer et al. (2009), Scholl et al. (2009), Reichold et al. (2010), Sicca et al. (2011), Sibille et al. (2014)

## Ca^2+^-ACTIVATED K^+^ CHANNELS

The calcium-activated K^+^ (K_Ca_) channels are highly conserved across species, and widely expressed in the human brain. The phylogenetic tree of K_Ca_ channels shows that they are made of two well distinct groups (Wei et al., 2005), the large conductance (BK; K_Ca_1.1), and the small/intermediate-conductance (SK/IK; K_Ca_2.1, K_Ca_2.2, K_Ca_2.3, K_Ca_3.1) K_Ca_ channels. With regard to gating mechanism, the Ca^2+^ sensitivity of SK/IKchannels is provided by tightly bound calmodulin (Xia et al., 1998; Fanger et al., 1999), in contrast to the direct binding of Ca^2+^ at specific internal sites on the channel protein of K_Ca_1.1 channels (Lee and Cui, 2010). Moreover, unlike the SK/IK channels, K_Ca_1.1 channels are also activated by voltage. In brain neurons, K_Ca_ channels are widely distributed in axons and presynaptic terminals (Knaus et al., 1996; Blank et al., 2004), often located close to voltage-gated Ca^2+^ channels Ca_v_ (Marrion and Tavalin, 1998). Ca^2+^ influx that results from neuronal excitation activates K_Ca_ channels, whose outward K^+^ flux contributes to terminate the action potential, establish the afterhyperpolarization (AHP) and close Ca_v_ channels. This negative feed-back control has been generally assumed to make K_Ca_ channels critical players in opposing repetitive firing and hyperexcitability typical of epileptic disorders. To date mutations only in the K_Ca_1.1 channel type have been clearly associated to both autism and epilepsy.

### K_**Ca**_1.1 – ChannelASD

The *KCNMA1* gene encodes for the α subunit of K_Ca_1.1 channel which possesses a tetrameric structure with four optional auxiliary β subunits (Sausbier et al., 2004). Channel activation requires both an increase in intracellular Ca^2+^ concentration and membrane depolarization (Storm, 1987; Robitaille et al., 1993). Thanks to its Ca^2+^- and voltage-dependence it negatively regulates Ca^2+^ entry and provides a damping mechanism for excitatory signals in many neuronal types. K_Ca_1.1 channels are expressed in the hippocampus, cortex and cerebellum where they contribute to both action potential repolarization and brief AHP (Lancaster and Nicoll, 1987; Robitaille and Charlton, 1992; Shao et al., 1999; Hu et al., 2001; Womack et al., 2009). Experimentally, the contribution of presynaptic *I_KCa1.1_* is not easily detectable under basal conditions (Storm, 1987). It can be unmasked by blocking voltage-gated K^+^ channels with 4-aminopyridine that broadens the presynaptic action potential. Under these conditions, blocking K_Ca_1.1 channels in CA1 with *iberiotoxin* (IbTX) causes further broadening of the presynaptic compound action potential, enhancement of synaptic transmission and reduced paired-pulse facilitation ratio (Hu et al., 2001). In cerebellar Purkinje neurons (PNs), the contribution of K_Ca_1.1 channel to the AHP phase is developmentally regulated with a greater contribution in immature neurons than in adult PNs (Womack et al., 2009). *In vivo*, deletion of the *Kcnma1* gene profoundly alters cerebellar function: K_Ca_1.1^-/-^ mice show an abnormal conditioned eye blink response, abnormal locomotion and pronounced lack of coordination (Sausbier et al., 2004). These changes are, in part, the direct consequence of a profound disinhibition of deep cerebellar nuclei (DCN). Indeed, while all the wild-type PNs discharge spontaneous APs at baseline, 50% of K_Ca_1.1^-/-^ PNs become quiescent. Since these silent neurons have a depolarized membrane potential, a depolarization block mediated by inactivated voltage-gated Na^+^ channels has been invoked as the underlying molecular mechanism (Sausbier et al., 2004). Thus, K_Ca_1.1 channels ablation dramatically lowers the overall discharge activity of PNs. The close correlation between the loss of the K_Ca_1.1 channel in PNs and the cerebellar deficit was further confirmed by the genesis of PN-K_Ca_1.1^-/-^ mice, where K_Ca_1.1 expression was specifically abolished in PNs (Chen et al., 2010). These animals recapitulate the motor deficits observed in K_Ca_1.1^-/-^ mice, although to a lesser extent and show, *in vivo*, a reduction of both simple spike (SS) and complex spike (CS) activity in PNs. Recall that SS is generated by afferent parallel fibers (PF) while CS is induced by climbing fibers (CF). In these mice, the olivo-cerebellar circuit is likely deregulated since reductions in PNs inhibitory inputs result in an enhancement of DCNs activity which, in turn, exert an excessive inhibition on downstream inferior olive (IO). Interestingly, the application of muscimol (a GABA_A_ receptor agonist) onto DCN partially restores CS activity (Chen et al., 2010).

In humans, the *KCNMA1 gain-of-function* mutation D434G has been found in patients suffering from K_Ca_1.1-*channelepsy* that is characterized by generalized epilepsy and paroxysmal dyskinesia (Du et al., 2005; D’Adamo et al., 2013). Expression studies indicated that the D434G mutant channel results in markedly greater macroscopic currents and single-channel open probability due to ~5-fold increase in Ca^2+^ sensitivity (Du et al., 2005). The enhancement of K_Ca_1.1 channel activity may lead to increased excitability by inducing rapid repolarization of action potentials, thus allowing neurons to fire at faster rates (Du et al., 2005). The possible role of K_Ca_1.1 channels in ASD phenotype has been highlighted by Laumonnier et al. (2006) who have identified both the substitution A138V and the *de novo* translocation *t*(9;10) (q23; q22) in the *KCNMA1* gene of two unrelated autistic patients (**Table 1**). The autistic patient carrying the substitution displayed epilepsy, impairments of reciprocal social interactions and communications skills, lack of spoken language and poor communicating gestures. The A138V substitution is located within the second exon of *KCNMA1* and changes a residue highly conserved in evolution from *C. elegans* to mammals. Sequence analysis showed that the substitution creates a cryptic splice donor site that likely impairs channel expression. The *de novo* translocation was found in a 6 year old boy, displaying typical symptoms of autism. The translocation separates the promoter of *KCNMA1* from the rest of the gene, leading to a non-functional allele and haploinsufficiency. In fact, the *KCNMA1* transcript levels (determined from patient’s lymphoblastoid cells) were halved. In addition, patch-clamp recordings from these cells revealed depolarized Vm, increased input resistance and halved IbTX-sensitive current (Laumonnier et al., 2006). Physiological K_Ca_1.1 channel dosage is crucial for network functionality. Indeed, either loss or gain of K_Ca_1.1 channel activity are both capable to unbalance cell excitability, markedly (Sausbier et al., 2004; Du et al., 2005; Chen et al., 2010). K_Ca_1.1 *loss-of-function* mutations likely alter pyramidal neurons excitability and result in impairment of neural networks in hippocampus, an area implicated in cognition, mood disorders, and ASD. However, these mutations may also affect cerebellar PNs excitability, development, learning and memory processes, suggesting that K_Ca_1.1 channels dysfunction may impact these crucial neurophysiological processes occurring within the cerebellum and result in the psychomotor development and cognition impairments of ASD (*see below*).

### K_**Ca**_1.1 – and SLACK – ChannelASD linked to Fragile X syndrome

Recently, K_Ca_1.1 channels have been implicated in ASD on a different ground, since their activity is regulated by FMRP, whose mutation produces FXS. We have mentioned previously that FMRP is mainly located postsynaptically, however, important pre-synaptic functions have been recently unmasked. FMRP is present in developing growth cones of axons (Antar et al., 2006), and it has been proposed to play pre-synaptic roles in neural wiring establishment (Hanson and Madison, 2007; Christie et al., 2009). Importantly, in pre-synaptic terminals FMRP regulates K_Ca_1.1 channel Ca^2+^ sensitivity via interactions with the channel’s regulatory β4 subunit (Deng et al., 2013). Loss of FMRP, as obtained in *Fmr1*-null mice, reduces K_Ca_1.1 Ca^2+^ sensitivity leading to smaller current, AP broadening and increased short-term plasticity (STP) in hippocampal CA3 pyramidal neurons (Deng et al., 2011, 2013; **Table 2**). The application of IbTX, a selective inhibitor of K_Ca_1.1 channels, reproduced in WT mice the effects observed in *Fmr1-*null mice (Deng et al., 2013). In addition, *Fmr1-*null mice displayed abnormal eye-blink conditioning, atypically elongated PNs spines and enhanced LTD at PF-PNs synapses, leading to reduced PNs response to PF stimuli. The enhancement in LTD induction found in *Fmr1-*null mice was reproduced in animals where the *Fmr1* gene was ablated specifically in PNs. This more direct evidence supports the notion that intrinsic PNs defects account for altered synaptic plasticity in the cerebellum of animals lacking FMRP and the crucial role that this brain structure may play in ASDs (Koekkoek et al., 2005).

**Table 2:** Indirect involvement of K^+^ channels in ASD.

Gene/channel	Neurophysiological role	Regulation by FMRP	Effect of FMRP loss at network level	Reference
***KCND2*/K_**v**_4.2**	See: **Table 1**.	– FMRP regulates K_v_4.2 mRNA translation.	– Higher K_v_4.2 expression in hippocampal neurons resulting in reduced LTP.	Kim and Hoffman (2008), Lee et al. (2011)
***KCNMA1*/K_**ca**_1.1(BK)**	See: **Table 1**.	– FMRP modulates Ca^2+^ sensitivity of channels via interactions with the regulatory β4 subunits.	– Excessive AP broadening during repetitive activity, enhanced pre-synaptic Ca^2+^ influx and abnormal increased STP.	Deng et al. (2011, 2013)
***KCNT1*/SLACK**	– Neuronal: contributes to the slow hyperpolarization that follows repetitive firing and regulates the rate of bursting.	– FMRP binds to the *Slack* cytoplasmic *C*-terminal and induces an increase in channel opening.	– Reduced *I_K(Na)_* component in MNTB neurons.	Bhattacharjee and Kaczmarek (2005), Brown et al. (2010)

Notably, FMRP can also bind to Na^+^-activated K^+^ channel *Slack*, and thus regulate its activity (Brown et al., 2010). This channel type is mainly expressed in the cortex, hippocampus, olfactory bulb and lateral MNTB neurons of the brainstem (Brown et al., 2008) where it contributes to the slow hyperpolarization that follows repetitive firing and regulates the rate of bursting (Bhattacharjee and Kaczmarek, 2005). At the single channel level, *Slack* generates a very high conductance (~180 pS) with frequent transitions to a relatively long-lived sub-state with a conductance about one-third that of the fully open state. The binding of FMRP to the cytoplasmic *C*-terminal of *Slack* induces a fast and dramatic effect on these channel gating properties, namely, the transition to the sub-states is almost completely abolished, and there is a marked increase in channel opening (Brown et al., 2010). In *Fmr1-KO* mice, whole-cell recording of lateral MNTB neurons shows a significantly reduced *I_K(Na)_* component compared to WT, consistent with a role for FMRP in regulating the activity of native *Slack* channels (Brown et al., 2010). Autosomal dominant nocturnal frontal lobe epilepsy (ADNFLE) is caused by mutations in the *CHRNA4*, *CHRNA2, CHRNB2,* or *KCNT1*(*Slack*) genes (Steinlein et al., 1995; De Fusco et al., 2000; Aridon et al., 2006; Heron et al., 2012). Interestingly, intellectual disability only occurs in those patients who carry mutations in *Slack* channels (Heron et al., 2012), further suggesting a role for this channel type in both epilepsy and cognitive disorders (*reviewed in* Kim and Kaczmarek, 2014).

## INWARDLY RECTIFYING K^+^ CHANNELS

Inwardly rectifying K^+^ (Kir) channels take their name from the greater conductance at potentials negative to *E*_K_, while at more positive values the outward flow of K^+^ ions is variably inhibited by cytoplasmic polyamines and Mg**^2+^**, by means of affinity dependent blockade. Indeed, the different sensitivity of Kir channels to polyamines and Mg**^2+^** dictates the degree of rectification from weak to strong (Matsuda et al., 1987; Lopatin et al., 1994; Lu and Mac Kinnon, 1994; Stanfield et al., 1994). Cells that possess large Kir conductance display a resting membrane potential (Vm) close to *E*_K_. Beside setting the Vm values, Kir channels of excitable cells play key roles in the regulation of action potential duration and excitability. Several Kir clones have been identified and classified into seven major subfamilies, Kir1.x-Kir7.x (Bond et al., 1994; Hibino et al., 2010). Structurally, a Kir subunit possesses two transmembrane domains separated by a pore-forming region, serving as the “ion-selectivity filter,” with both the *N*- and *C*-termini residing inside the cell. Four subunits are assembled as both homotetramers and heterotetramers (Hibino et al., 2010).

### Kir2.1 – ChannelASD

*KCNJ2* encodes for Kir2.1 channels that possess a conductance of ~30 pS and generate macroscopic currents with strong rectification properties. High levels of *KCNJ2* transcript are found in the brain, heart and skeletal muscle. In the brain, Kir2.1 is predominantly expressed in the hippocampus, caudate, putamen, nucleus accumbens, and to lower levels in habenula and amygdala (Karschin et al., 1996), where it contributes to control neuronal excitability. In particular, the amplitude of Kir2.1 currents is small in young dentate granule neurons (DGCs) and increases ~3-fold in mature DGCs to optimize their excitability. Thus, Kir2.1 channels play an important role in DGCs firing properties during development (Mongiat et al., 2009). Kir2.1 is also expressed in astrocytes and, in combination with Kir4.1, controls astrocyte-mediated K^+^ buffering (*see below*; Bordey and Sontheimer, 1998; Jabs et al., 2008; Chever et al., 2010). *Loss-of-function* mutations in the *KCNJ2* gene are responsible for the rare Andersen-Tawil syndrome (OMIM 170390) a condition characterized by long QT-syndrome, cardiac arrhythmia, skeletal abnormalities, periodic paralysis, mood disorders, and seizures (Haruna et al., 2007; Chan et al., 2010). Conversely, Kir2.1 *gain-of-function* mutations segregate with SQT3 syndrome (OMIM 609622), another cardiac disorder characterized by QT shortening, ventricular tachyarrhythmias and atrial fibrillation. Indeed, in the heart Kir2.1 is a component of the inward-rectifier current IK1, which provides substantial repolarizing current during the terminal repolarization phase of the cardiac action potential and is the primary conductance controlling the diastolic membrane potential (Hutter and Noble, 1960; Sanguinetti and Tristani-Firouzi, 2000).

Recently, we reported on the identification of a new K346T heterozygous mutation in the *KCNJ2* of monozygotic twins displaying autism and epilepsy *in cis* with the previously detected R18Q variant in *KCNJ10* (Sicca et al., 2011; Ambrosini et al., 2014; **Table 1**). The twins also showed an electrocardiogram (ECG) with a markedly short repolarization time and conspicuously narrow and peaked T waves (QTc interval, 331 ms). K346T expression resulted in larger homozygous and heterozygous K^+^ currents due to increased surface expression of the channel in oocytes, HEK293 and glial-like cells. Functionally, several deleterious defects were also described for this novel *KCNJ2* variant which: (i) enhanced the channel’s stability at the plasma membrane; (ii) reduced protein ubiquitylation and degradation; (iii) altered protein compartmentalization in lipid rafts, by targeting more channels to cholesterol-poor domains; (iv) reduced interactions with caveolin 2. All these molecular mechanisms contributed to causing Kir2.1 *gain-of-function*. Notably, the mutation promotes the surface expression of the channels particularly at end-feet, filopodia-like structures and cell–cell contacts. These structures are essential for astrocyte-mediated K^+^ siphoning through Kir2.1, Kir4.1, and Kir5.1 channels, all of which could be influenced by the K346T mutation. Given that Kir2 channels also contribute to regulating neuronal excitability, cell differentiation, synaptic plasticity and wiring, their dysfunction may impact these crucial neurophysiological processes. Therefore, this study proposed that genetically induced Kir2.1 defects, beside causing SQT3 syndrome, may possibly result in functional impairment of neural networks where this channel type resides and contributes to ASDs pathogenesis (Ambrosini et al., 2014).

### Kir4.1 – ChannelASD

The *KCNJ10* gene encodes for Kir4.1 channels (Bond et al., 1994), which forms homomeric channels or polymerize with Kir5.1 (*KCNJ16*) to form heterotetramers (Pessia et al., 1996) highly sensitive to pH (Tucker et al., 2000; Pessia et al., 2001; Casamassima et al., 2003; D’Adamo et al., 2011b). Kir4.1 is mainly expressed in *locus coeruleus* neurons, oligodendrocytes and astrocytes surrounding synapses and blood vessels from cortex, thalamus, hippocampus, and brainstem (Takumi et al., 1995; Higashi et al., 2001; D’Adamo et al., 2011b). In these cells, Kir4.1 channels generate high K^+^ permeability driving the membrane potential near E_K_ values. During excitatory synaptic activity, astrocytes surrounding synapses slowly depolarize because of K^+^ influx across their membranes (Orkand et al., 1966). This prolonged inward K^+^ current is mediated mainly by Kir4.1 and is synchronized with synaptic and spiking activity (Sibille et al., 2014). Thanks to the functional coupling between neurons and astrocytes the excess of extracellular K^+^ ions, resulting from intense neuronal firing, is taken up by astrocytes by flowing through Kir4.1 channels. Then, K^+^ ions are transferred toward sites where their concentration is kept at low levels by the gap-junction cell-syncytium (Kuﬄer and Nicholls, 1966). By this mechanism, known as “*K^+^ siphoning,*” the concentration of extracellular K^+^ in CNS is strictly ruled about 2–3 mM, avoiding neuronal after-discharges and depolarization block. In the mouse hippocampus, Kir4.1 is significantly up-regulated between P3 and P12 (Seifert et al., 2009) and its expression is closely related to cell differentiation. In fact, Kir4.1 channels are absent in immature proliferating cells of the glia, and its progressive expression is linked to astrocytes hyperpolarization, differentiation and exit from cell cycle (from G2/M to G0/G1; Higashimori and Sontheimer, 2007). Genetic ablation of Kir4.1 in rodents results in severe motor deficits, hypomyelination in the spinal cord, severe spongiform vacuolation, astrocytes with immature morphology and premature death before P24 (Neusch et al., 2001). Conditional ablation of Kir4.1, exclusively in astrocytes, resulted in a mouse phenotype characterized by pronounced body tremor, lethargy, ataxia as well as visual placing deficiency and stress-induced seizures (Djukic et al., 2007). In humans, *KCNJ10 loss-of-function* mutations segregate with the EAST or SeSAME syndrome, a rare autosomic recessive disorder characterized by epilepsy, ataxia, sensorineural deafness, and tubulopathy. The patients develop tonic–clonic seizures in infancy followed by motor delay, speech and mental retardation (Bockenhauer et al., 2009; Scholl et al., 2009; Reichold et al., 2010). In line with the pathophysiological relationship between the Kir4.1 channel impairment and both epilepsy and developmental disorders, we investigated the frequency of *KCNJ10* mutations in several children with cryptogenic epilepsy and autism spectrum traits. We found two inherited heterozygous *KCNJ10* mutations at residues highly conserved in mammalian and vertebrate orthologs: R18Q in two identical twins and V84M in another child. The expression of mutant channels in *Xenopous laevis* oocytes resulted in current/voltage relationships with greater amplitudes than WT for both mutations, suggesting a *gain-of-function* effect. Patch-clamp single-channel recordings revealed that the V84M mutation increased the unit conductance ~1.5 fold compared to WT. The R18Q mutation altered neither the unit conductance nor the single channel parameters, yet increased the channel surface expression. Clinically, the two identical twins showed impaired social interaction, sleep difficulties, hypotonia and both exhibited epileptic spasms occurring within the same 24 h period. Other symptoms, typical of ASD, included clumsiness, absence of speech, severe disorder of social interaction, stereotypies, repetitive behaviors, symptoms of anxiety, depression, obsessive compulsive disorder and intellectual disability (IQ: 58). The child harboring the V84M mutation showed normal psychomotor development until 12 months of age, when ASD symptoms such as poor social gaze, no response to name, absence of language development, and withdrawal behaviors became evident. At the age of six, he experienced complex partial seizures. EEG recordings showed synchronous and asynchronous paroxysmal abnormalities over frontal regions in both hemispheres, tending to spread (Sicca et al., 2011; **Table 1**). Co-occurrence of epilepsy and ASD in patients harboring *KCNJ10 gain-of-function* mutations suggests that dysfunction in the astrocytic-dependent K^+^ buffering may be a common mechanism contributing to seizures as well as the core behavioral features of ASD. Kir4.1 *gain-of-function* mutations may lead to faster and larger influx of K^+^ into astrocytes, during intense neuronal activity, resulting in membrane depolarization and higher intracellular Ca^2+^ elevations in these cells. Ca^2+^ elevations in astrocytes are associated with gliotransmitters’ release, such as glutamate and D-serine that trigger discharges in neurons and promote local neuronal synchrony and epileptic activity (Steinhäuser et al., 2015). Abnormal synaptic function is the common basis for ASDs. Inasmuch as the activity of many thousands of synapses is controlled by a single astrocyte and this cell type makes up 90% of all human brain cells, defective astrocyte-dependent CNS development and regulation of K^+^ homeostasis in the brain represent original mechanistic hypotheses linking the allelic variations we have identified in *KCNJ2* and *KCNJ10* and ASD.

## ChannelASD-CHANNELEPSY PHENOTYPE

Mutations in K^+^ channels have been widely associated with several forms of channelepsies (D’Adamo et al., 2013). The analysis of the literature performed here shows that in nearly all cases affected by K^+^ channelASDs, epilepsy is always comorbid. The degree of clinical and genetic overlap between K^+^ channelASDs and some K^+^ channelepsies, suggests that a subcategory named *channelASD-channelepsy phenotype* may be distinguished. Indeed, this scenario could be envisioned for some other channel types, playing significant roles in brain development, behavior and cognition. An effective example in this direction comes from Dravet’s syndrome (DS), a devastating neurodevelopmental disorder (Catterall, 2000; De Jonghe, 2011). DS is characterized by comorbidity of epilepsy and psychiatric disorders, and linked to Nav1.1(*SCN1A*) channel dysfunction. Indeed, together with early-life intractable seizures, patients develop devastating psychomotor, cognitive and behavioral deficits persisting through adulthood (Bender et al., 2012). Thus, *SCN1A* has been suggested as a candidate gene for ASD (Weiss et al., 2003). The implication of this gene was confirmed by whole-exome sequencing of sporadic ASD cases that revealed a missense mutation in a severely affected patient with evidence of early onset, language delay, epilepsy, and mild intellectual disability. This mutation leads to replacement of the highly conserved proline 1894 with lysine, and is predicted to be functionally deleterious (O’Roak et al., 2011). *Scn1a^-/+^* rodents recapitulate DS (Yu et al., 2006; Kalume et al., 2007). The loss of Nav1.1 channels, which is mostly expressed in GABA*ergic* interneurons, leads to a marked reduction in the inhibitory activity of these cells that reverberates onto prefrontal and hippocampal pyramidal neurons, which consequently fire at higher frequencies (Han et al., 2012). Remarkably, these mice show epilepsy and a spectrum of behavioral abnormalities characteristic of ASD such as hyperactivity, anxiety, altered behavioral interaction and deficits in context-dependent spatial memory (Yu et al., 2006; Han et al., 2012). Thus, it could be possible to hypothesize that distinct channel-dependent autism/epilepsy cases share a common etiopathological denominator, namely an excitatory-inhibitory imbalance generating a non-permissive substrate for the physiological development of the proper cognitive and behavioral skills, resulting in *channelASD-channelepsy phenotype.*

## CONCLUDING REMARKS

Autism spectrum disorders are amongst the most common neuropsychiatric diseases (Blumberg et al., 2013) that show increasing prevalence over the past years and no discrimination in terms of ethnicity, family income or educational levels. However, how much of this increase is due to a broader definition of ASD and better efforts in diagnosis or an effective increased incidence of the disorders, is unclear. The number of ASDs cases that could be accounted for a channel-dependent pathogenesis and classified in the *channelASD* subgroup, remains to be established. Inasmuch as ~70 million people suffer from ASD and nearly 500 ion channel proteins are encoded by the human genome, the worldwide number of *channelASD* cases might not be negligible.

Despite the efforts made, the etiology of ASD remains largely elusive because many cases arise from a mixture of multiple environmental and genetic factors. In addition, the modalities by which the combinations of different genetic variations contribute to the overall risk to develop the disease are mostly obscure. Nevertheless, a mounting body of evidence indicates that ion channel dysfunction may well enhance autism susceptibility (Schmunk and Gargus, 2013) also when other contributing alleles are co-inherited. Direct and indirect defects in K^+^ channels have been implicated in ASDs pathogenesis, likely altering crucial neural network processes in several brain areas including the cerebellum, a structure that emerges as critically involved in determining the core features of ASDs. Abnormal synaptic transmission and dendritic spine pathology play crucial roles in ASDs. Notably, the activity of many thousands synapses is controlled by a single astrocyte. Thus, aberrant astrocyte-dependent synaptic functions and CNS development, induced by defective ion channels, represent an interesting causative hypotheses for ASDs (D’Adamo et al., 2011a; Sicca et al., 2011; Ambrosini et al., 2014). Undeniably, the high heterogeneity of ASDs makes the solution of the “*autism puzzle*” an extremely difficult task. Notwithstanding, to ensure the best possible outcomes for children affected by this devastating disease, comprehensive socio–economic policies and coordinated scientific efforts are required as a matter of urgency.

## Conflict of Interest Statement

The authors declare that the research was conducted in the absence of any commercial or financial relationships that could be construed as a potential conflict of interest.

## References

[B1] Ambrosini E. Sicca F. Brignone M. S. D’Adamo M. C. Napolitano C. Servettini I. (2014). Genetically induced dysfunctions of Kir2.1 channels: implications for short QT3 syndrome and autism-epilepsy phenotype. *Hum. Mol. Genet.* 23 4875–4886 10.1093/hmg/ddu201 24794859 PMC4140467

[B2] Antar L. N. Li C. Zhang H. Carroll R. C. Bassell G. J. (2006). Local functions for FMRP in axon growth cone motility and activity-dependent regulation of filopodia and spine synapses. *Mol. Cell. Neurosci.* 32 37–48 10.1016/j.mcn.2006.02.001 16631377

[B3] Aridon P. Marini C. Di Resta C. Brilli E. De Fusco M. Politi F. (2006). Increased sensitivity of the neuronal nicotinic receptor alpha 2 subunit causes familial epilepsy with nocturnal wandering and ictal fear. *Am. J. Hum. Genet.* 79 342–350 10.1086/506459 16826524 PMC1559502

[B4] Bahring R. Boland L. M. Varghese A. Gebauer M. Pongs O. (2001). Kinetic analysis of open- and closed-state inactivation transitions in human Kv4.2 A-type potassium channels. *J. Physiol.* 535 65–81 10.1111/j.1469-7793.2001.00065.x 11507158 PMC2278757

[B5] Bahring R. Covarrubias M. (2011). Mechanisms of closed-state inactivation in voltage-gated ion channels. *J. Physiol.* 589 461–479 10.1113/jphysiol.2010.191965 21098008 PMC3055536

[B6] Barghaan J. Bahring R. (2009). Dynamic coupling of voltage sensor and gate involved in closed-state inactivation of kv4.2 channels. *J. Gen. Physiol.* 133 205–224 10.1085/jgp.200810073 19171772 PMC2638201

[B7] Barnwell L. F. Lugo J. N. Lee W. L. Willis S. E. Gertz S. J. Hrachovy R. A. (2009). Kv4.2 knockout mice demonstrate increased susceptibility to convulsant stimulation. *Epilepsia* 50 1741–1751 10.1111/j.1528-1167.2009.02086.x 19453702 PMC2925051

[B8] Bechara E. G. Didiot M. C. Melko M. Davidovic L. Bensaid M. Martin P. (2009). A novel functionfor fragile X mental retardation protein in translational activation. *PLoS Biol.* 7:e16 10.1371/journal.pbio.1000016 PMC2628407 19166269

[B9] Bender A. C. Morse R. P. Scott R. C. Holmes G. L. Lenck- Santini P. P. (2012). SCN1A mutations in Dravet syndrome: impact of interneuron dysfunction on neural networks and cogni- tive outcome. *Epilepsy Behav.* 23 177–186 10.1016/j.yebeh.2011.11.022 22341965 PMC3307886

[B10] Bhattacharjee A. Kaczmarek L. K. (2005). For K^+^ channels, Na^+^ is the new Ca2^+^. *Trends Neurosci.* 28 422–428 10.1016/j.tins.2005.06.003 15979166

[B11] Birnbaum S. G. Varga A. W. Yuan L. L. Anderson A. E. Sweatt J. D. Schrader L. A. (2004). Structure and function of Kv4-family transient potassium channels. *Physiol. Rev.* 84 803–833 10.1152/physrev.00039.2003 15269337

[B12] Blank T. Nijholt I. Kye M. J. Spiess J. (2004). Small conductance Ca2+- activated K+ chan- nels as targets of CNS drug development. *Curr. Drug Targets CNS Neurol. Disord.* 3 161–167 10.2174/1568007043337472 15180477

[B13] Blumberg S. J. Bramlett M. D. Kogan M. D. Schieve L. A. Jones J. R. Lu M. C. (2013). *Changes in Prevalence of Parent-Reported Autism Spectrum Disorder in School-Aged U.S. Children: 2007 to 2011–2012*. Hyattsville, MD: National Center for Health Statistics. 24988818

[B14] Bockenhauer D. Feather S. Stanescu H. C. Bandulik S. Zdebik A. A. Reichold M. (2009). Epilepsy, ataxia, sensorineural deafness, tubulopathy, and KCNJ10 mutations. *N. Engl. J. Med.* 360 1960–1970 10.1056/NEJMoa0810276 19420365 PMC3398803

[B15] Bond C. T. Pessia M. Xia X. M. Lagrutta A. Kavanaugh M. P. Adelman J. P. (1994). Cloning and expression of a family of inward rectifier potassium channels. *Receptors Channels* 2 183–191. 7874445

[B16] Bordey A. Sontheimer H. (1998). Properties of human glial cells associated with epileptic seizure foci. *Epilepsy Res.* 32 286–303 10.1016/S0920-1211(98)00059-X 9761328

[B17] Brown D. A. Adams P. R. (1980). Muscarinic suppression of a novel voltage-sensitive K^+^ current in a vertebrate neurone. *Nature* 283 673–676 10.1038/283673a0 6965523

[B18] Brown M. R. Kronengold J. Gazula V. R. Chen Y. Strumbos J. G. Sigworth F. J. (2010). Fragile X mental retardation protein controls gating of the sodium-activated potassium channel slack. *Nat. Neurosci.* 13 819–821 10.1038/nn.2563 20512134 PMC2893252

[B19] Brown M. R. Kronengold J. Gazula V. R. Spilianakis C. G. Flavell R. A. Von Hehn C. A. (2008). Amino-termini isoforms of the slack K^+^ channel, regulated by alternative promoters, differentially modulate rhythmic firing and adaptation. *J. Physiol.* 586 5161–5179 10.1113/jphysiol.2008.160861 18787033 PMC2652154

[B20] Buzsáki G. Draguhn A. (2004). Neuronal oscillations in cortical networks. *Science* 304 1926–1929 10.1126/science.1099745 15218136

[B21] Casamassima M. D’Adamo M. C. Pessia M. Tucker S. J. (2003). Identification of a heteromeric interaction which influences the rectification gating and pH-sensitivity of Kir41/ Kir51 potassium channels. *J. Biol. Chem.* 278 43533–43540 10.1074/jbc.M306596200 12923169

[B22] Catterall W. A. (2000). From ionic currents to molecular mechanisms: the structure and function of voltage-gated sodium channels. *Neuron* 26 13–25 10.1016/S0896-6273(00)81133-2 10798388

[B23] Chan H. F. Chen M. L. Su J. J. Ko L. C. Lin C. H. Wu R. M. (2010). A novel neuropsychiatric phenotype of KCNJ2 mutation in one Taiwanese family with Andersen-Tawil syndrome. *J. Hum. Genet.* 55 186–188 10.1038/jhg.2010.2 20111058

[B24] Chandy K. G. Gutman G. A. (1993). Nomenclature form a mammalian potassium channel genes. *Trends Pharmacol. Sci.* 14 434 10.1016/0165-6147(93)90181-I 8122319

[B25] Chen X. Kovalchuk Y. Adelsberger H. Henning H. A. Sausbier M. Wietzorrek G. (2010). Disruption of the olivo-cerebellar circuit by Purkinje neuron-specific ablation of BK channels. *Proc. Natl. Acad. Sci. U.S.A.* 107 12323–12328 10.1073/pnas.1001745107 20566869 PMC2901450

[B26] Chen X. Yuan L. L. Zhao C. Birnbaum S. G. Frick A. Jung W. E. (2006). Deletion of Kv4.2 gene eliminates dendritic A-type K^+^ current and enhances induction of long-term potentiationin hippocampal CA1 pyramidal neurons. *J. Neurosci.* 26 12143–12151 10.1523/JNEUROSCI.2667-06.2006 17122039 PMC6675426

[B27] Chever O. Djukic B. McCarthy K. D. Amzica F. (2010). Implication of Kir4.1 channel in excess potassium clearance: an in vivo study on anesthetized glial-conditional Kir4.1 knock-out mice. *J. Neurosci.* 30 15769–15777 10.1523/JNEUROSCI.2078-10.2010 21106816 PMC6633770

[B28] Christie S. B. Akins M. R. Schwob J. E. Fallon J. R. (2009). The FXG: a presynaptic fragile X granule expressed in a subset of developing brain circuits. *J. Neurosci.* 29 1514–1524 10.1523/JNEUROSCI.3937-08.2009 19193898 PMC2746438

[B29] Clarke D. F. Roberts W. Daraksan M. Dupuis A. McCabe J. Wood H. (2005). The prevalence of autistic spectrum disorder in children surveyed in a tertiary care epilepsy clinic. *Epilepsia* 46 1970–1977 10.1111/j.1528-1167.2005.00343.x 16393164

[B30] Covarrubias M. Bhattacharji A. De Santiago-Castillo J. A. Dougherty K. Kaulin Y. A. Na-Phuket T. R. (2008). The neuronal Kv4 channel complex. *Neurochem. Res.* 33 1558–1567 10.1007/s11064-008-9650-8 18357523 PMC5833991

[B31] D’Adamo M. C. Catacuzzeno. L. Di Giovanni G. Franciolini F. Pessia M. (2013). K^+^ channelepsy: progress in the neurobiology of potassium channels and epilepsy. *Front. Cell Neurosci.* 7:134 10.3389/fncel.2013.00134 PMC3772396 24062639

[B32] D’Adamo M. C. Di Giovanni G. Pessia M. (2014). “Animal models of episodic ataxia Type 1 (EA1),” in *Movement Disorders: Genetics and Models* 2nd Edn ed. LeDoux M. S. (New York, NY: Academic Press Inc./Elsevier Science Publishing Co. Inc.).

[B33] D’Adamo M. C. Moro F. Imbrici P. Martino D. Roscini M. Santorelli F. M. (2011a). The emerging role of the inwardly rectifying K^+^ channels in autism spectrum disorders and epilepsy. *Malta Med. J.* 23 10–14.

[B34] D’Adamo M. C. Shang L. Imbrici P. Brown S. D. M. Pessia M. Tucker S. (2011b). Genetic inactivation of Kcnj16 identifies Kir 5.1 as an important determinant of neuronal PCO2/pH sensitivity. *J. Biol. Chem.* 286 192–198 10.1074/jbc.M110.189290 21047793 PMC3012974

[B35] De Fusco M. Becchetti A. Patrignani A. Annesi G. Gambardella A. Quattrone A. (2000). The nicotinic receptor beta 2 subunit is mutant in nocturnal frontal lobe epilepsy. *Nat. Genet.* 26 275–276 10.1038/81566 11062464

[B36] De Jonghe P. (2011). Molecular genetics of Dravet syndrome. *Dev. Med. Child Neurol.* 53(Suppl. 2) 7–10 10.1111/j.1469-8749.2011.03965.x 21504425

[B37] Deng P. Y. Rotman Z. Blundon J. A. Cho Y. Cui J. Cavalli V. (2013). FMRP regulates neurotransmitter release and synaptic information transmission by modulating action potential duration via BK channels. *Neuron* 77 696–711 10.1016/j.neuron.2012.12.018 23439122 PMC3584349

[B38] Deng P. Y. Sojka D. Klyachko V. A. (2011). Abnormal presynaptic short term plasticity and information processing in a mouse model of fragile X syndrome. *J. Neurosci.* 31 10971–10982 10.1523/JNEUROSCI.2021-11.2011 21795546 PMC6623101

[B39] Djukic B. Casper K. B. Philpot B. D. Chin L. S. McCarthy K. D. (2007). Conditional knock-out of Kir4.1 leads to glial membrane depolarization, inhibition of potassium and glutamate uptake and enhanced short-term synaptic potentiation. *J. Neurosci.* 27 11354–11365 10.1523/JNEUROSCI.0723-07.2007 17942730 PMC6673037

[B40] Du W. Bautista J. F. Yang H. Diez-Sampedro A. You S.-A. Wang L. (2005). Calcium-sensitive potassium channelopathy in human epilepsy and paroxysmal movement disorder. *Nat. Genet.* 37 733–738 10.1038/ng1585 15937479

[B41] Eijkelkamp N. Linley J. E. Baker M. D. Minett M. S. Cregg R. Werdehausen R. (2012). Neurological perspectives on voltage-gated sodium channels. *Brain* 135 2585–2612 10.1093/brain/aws225 22961543 PMC3437034

[B42] Fanger C. M. Ghanshani S. Logsdon N. J. Rauer H. Kalman K. Zhou J. (1999). Calmodulin mediates calcium-dependent activation of the intermediate con ductance KCa channel, IKCa1. *J. Biol. Chem.* 274 5746–5754 10.1074/jbc.274.9.5746 10026195

[B43] Fu Y. H. Kuhl D. P. Pizzuti A. Pieretti M. Sutcliffe J. S. Richards S. (1991). Variation of the CGG repeat at the fragile X site results in genetic instability: resolution of the Sherman paradox. *Cell* 67 1047–1058 10.1016/0092-8674(91)90283-5 1760838

[B44] Garber K. B. Visootsak J. Warren S. T. (2008). Fragile X syndrome. *Eur. J. Hum. Genet.* 16 666–672 10.1038/ejhg.2008.61 18398441 PMC4369150

[B45] Gebauer M. Isbrandt D. Sauter K. Callsen B. Nolting A. Pongs O. (2004). N-type inactivation features of Kv4.2 channel gating. *Biophys. J.* 86 210–223 10.1016/S0006-3495(04)74097-7 14695263 PMC1303783

[B46] Gilling M. Rasmussen H. B. Calloe K. Sequeira A. F. Baretto M. Oliveira G. (2013). Dysfunction of the heteromeric KV7.3/ KV7.5 potassium channel is associated with autism spectrum disorders. *Front. Genet.* 4:54 10.3389/fgene.2013.00054 PMC3627139 23596459

[B47] Han S. Tai C. Westenbroek R. E. Yu F. H. Cheah C. S. Potter G. B. (2012). Autistic-like behaviour in Scn1a+/- mice and rescue by enhanced GABA-mediated neurotransmission. *Nature* 489 385–390 10.1038/nature11356 22914087 PMC3448848

[B48] Hanson J. E. Madison D. V. (2007). Presynaptic FMR1 genotype influences the degree of synaptic connectivity in a mosaic mouse model of fragile X syndrome. *J. Neurosci.* 27 4014–4018 10.1523/JNEUROSCI.4717-06.2007 17428978 PMC6672544

[B49] Haruna Y. Kobori A. Makiyama T. Yoshida H. Akao M. Doi T. (2007). Genotype-phenotype correlations of KCNJ2 mutations in Japanese patients with Andersen-Tawil syndrome. *Hum. Mutat.* 28 208 10.1002/humu.9483 17221872

[B50] Heron S. E. Smith K. R. Bahlo M. Nobili L. Kahana E. Licchetta L. (2012). Missense mutations in the sodium-gated potassium channel gene KCNT1 cause severe autosomal dominant nocturnal frontal lobe epilepsy. *Nat. Genet.* 44 1188–1190 10.1038/ng.2440 23086396

[B51] Hibino H. Inanobe A. Furutani K. Murakami S. Findlay I. Kurachi Y. (2010). Inwardly rectifying potassium channels, their structure, function, and physiological roles. *Physiol. Rev.* 90 291–366 10.1152/physrev.00021.2009 20086079

[B52] Higashi K. Fujita A. Inanobe A. Tanemoto M. Doi K. Kubo T. (2001). An inwardly rectifying K^+^ channel Kir4.1 expressed in astrocytes surrounds synapses and blood vessels in brain. *Am. J. Physiol. Cell Physiol.* 281 C922–C931. 11502569 10.1152/ajpcell.2001.281.3.C922

[B53] Higashimori H. Sontheimer H. (2007). Role of Kir4.1 channels in growth control of glia. *Glia* 55 1668–1679 10.1002/glia.20574 17876807 PMC2040118

[B54] Hou L. Antion M. D. Hu D. Spencer C. M. Paylor R. Klann E. (2006). Dynamic translational and proteasomal regulation of fragile X mental retardation protein controls mGluR-dependent long-term depression. *Neuron* 51 441–454 10.1016/j.neuron.2006.07.005 16908410

[B55] Hu H. Shao L. R. Chavoshy S. Gu N. Trieb M. Behrens R. (2001). Presynaptic Ca2^+^-activated K^+^ channels in glutamatergic hippocampal terminals and their role in spike repolarization and regulation of transmitter release. *J. Neurosci.* 21 9585–9597. 11739569 10.1523/JNEUROSCI.21-24-09585.2001 PMC6763057

[B56] Huber K. M. Gallagher S. M. Warren S. T. Bear M. F. (2002). Altered synaptic plasticity in a mouse model of fragile X mental retardation. *Proc. Natl. Acad. Sci. U.S.A.* 99 7746–7750 10.1073/pnas.122205699 12032354 PMC124340

[B57] Huber K. M. Kayser M. S. Bear M. F. (2000). Role for rapid dendritic protein synthesis in hippocampal mGluR-dependent long-term depression. *Science* 288 1254–1257 10.1126/science.288.5469.1254 10818003

[B58] Hutter O. F. Noble D. (1960). Rectifying properties of heart muscle. *Nature* 188 495 10.1038/188495a0 13717088

[B59] Jabs R. Seifert G. Steinhäuser C. (2008). Astrocytic function and its alteration in the epileptic brain. *Epilepsia* 49 3–12 10.1111/j.1528-1167.2008.01488.x 18226167

[B60] Jerng H. H. Dougherty K. Covarrubias M. Pfaffinger P. J. (2009). A novel N-terminal motif of dipeptidyl peptidase-like proteins produces rapid inactivation of KV4.2 channels by a pore-blocking mechanism. *Channels (Austin)* 3 448–461 10.4161/chan.3.6.10216 19901547 PMC3607289

[B61] Jerng H. H. Kunjilwar K. Pfaffinger P. J. (2005). Multiprotein assembly of Kv4.2, KChIP3 and DPP10 produces ternary channel complexes with ISA-like properties. *J. Physiol.* 568 767–788 10.1113/jphysiol.2005.087858 16123112 PMC1464192

[B62] Jerng H. H. Pfaffinger P. J. Covarrubias M. (2004). Molecular physiology and modulation of somatodendritic A-type potassium channels. *Mol. Cell. Neurosci.* 27 343–369 10.1016/j.mcn.2004.06.011 15555915

[B63] Jorde L. B. Hasstedt S. J. Ritvo E. R. Mason-Brothers A. Freeman B. J. Pingree C. (1991). Complex segregation analysis of autism. *Am. J. Hum. Genet.* 49 932–938. 1928098 PMC1683259

[B64] Jung S. C. Kim J. Hoffman D. A. (2008). Rapid, bidirectional remodeling of synaptic NMDA receptor subunit composition by A-type K^+^ channel activity in hippocampal CA1 pyramidal neurons. *Neuron* 60 657–671 10.1016/j.neuron.2008.08.029 19038222 PMC2637039

[B65] Kalume F. Yu F. H. Westenbroek R. E. Scheuer T. Catterall W. A. (2007). Reduced sodium current in Purkinje neurons from Nav1.1 mutant mice: implications for ataxia in severe myoclonic epilepsy in infancy. *J. Neurosci.* 27 11065–74 10.1523/JNEUROSCI.2162-07.2007 17928448 PMC6672849

[B66] Karschin C. Dissmann E. Stuhmer W. Karschin A. (1996). IRK(1-3) and GIRK(1-4) inwardly rectifying K^+^ channel mRNAs are differentially expressed in the adult rat brain. *J. Neurosci.* 16 3559–35570. 8642402 10.1523/JNEUROSCI.16-11-03559.1996 PMC6578832

[B67] Kim E. Hoffman D. A. (2012). Dynamic regulation of synaptic maturation state by voltage-gated A-type K^+^ channels in CA1 hippocampal pyramidal neurons. *J. Neurosci.* 32 14427–14432 10.1523/JNEUROSCI.2373-12.2012 23055512 PMC3489019

[B68] Kim G. E. Kaczmarek L. K. (2014). Emerging role of the KCNT1 Slack channel in intellectual disability. *Front. Cell Neurosci.* 8:209 10.3389/fncel.2014.00209 PMC4112808 25120433

[B69] Kim J. Hoffman D. A. (2008). Potassium channels: newly found players in synaptic plasticity. *Neuroscientist* 14 276–286 10.1177/1073858408315041 18413784 PMC2665047

[B70] Kim J. Jung S. C. Clemens A. M. Petralia R. S. Hoffman D. A. (2007). Regulation of dendritic excitability by activity-dependent trafficking of the A-type K^+^ channel subunit Kv4.2 in hippocampal neurons. *Neuron* 54 933–947 10.1016/j.neuron.2007.05.026 17582333 PMC1950443

[B71] Knaus H. G. Schwarzer C. Koch R. O. Eberhart A. Kaczorowski G. J. Glossmann H. (1996). Distribution of high-conductance Ca2^+^-activated K^+^ channels in rat brain: targeting to axons and nerve terminals. *J. Neurosci.* 16 955–963. 8558264 10.1523/JNEUROSCI.16-03-00955.1996 PMC6578788

[B72] Koekkoek S. K. Yamaguchi K. Milojkovic B. A. Dortland B. R. Ruigrok T. J. Maex R. (2005). Deletion of FMR1 in Purkinje cells enhances parallel fiber LTD, enlarges spines, and attenuates cerebellar eyelid conditioning in Fragile X syndrome. *Neuron* 47 339–352 10.1016/j.neuron.2005.07.005 16055059

[B73] Krawczun M. S. Jenkins E. C. Brown W. T. (1985). Analysis of the fragile-X chromosome: localization and detection of the fragile site in high resolution preparations. *Hum. Genet.* 69 209–211 10.1007/BF00293026 4038969

[B74] Kubisch C. Schroeder B. C. Friedrich T. Lutjohann B. El-Amraoui A. Marlin S. (1999). KCNQ4, a novel potassium channel expressed in sensory outer hair cells, is mutated in dominant deafness. *Cell* 96 437–446 10.1016/S0092-8674(00)80556-5 10025409

[B75] Kuﬄer S. W. Nicholls J. G. (1966). The physiology of neuroglial cells. *Ergeb. Physiol.* 57 1–90 10.1007/BF02259903 5330861

[B76] Lancaster B. Nicoll R. A. (1987). Properties of two calcium-activated hyperpolarizations in rat hippocampal neurones. *J. Physiol.* 389 187–203 10.1113/jphysiol.1987.sp016653 2445972 PMC1192077

[B77] Laumonnier F. Roger S. Guérin P. Molinari F. M’rad R. Cahard D. (2006). Association of a functional deficit of the BKCa channel, a synaptic regulator of neuronal excitability, with autism and mental retardation. *Am. J. Psychiatry* 163 1622–1629 10.1176/appi.ajp.163.9.1622 16946189

[B78] Lauterborn J. C. Rex C. S. Kramár E. Chen L. Y. Pandyarajan V. Lynch G. (2007). Brain-derived neurotrophic factor rescues synaptic plasticity in a mouse model of fragile X syndrome. *J. Neurosci.* 27 10685–10694 10.1523/JNEUROSCI.2624-07.2007 17913902 PMC6672822

[B79] Lavelle T. A. Weinstein M. C. Newhouse J. P. Munir K. Kuhlthau K. A. Prosser L. A. (2014). Economic burden of childhood autism spectrum disorders. *Pediatrics* 133:e520 10.1542/peds.2013-0763 PMC7034397 24515505

[B80] Lee H. Y. Ge W. P. Huang W. He Y. Wang G. X. Rowson-Baldwin A. (2011). Bidirectional regulation of dendritic voltage-gated potassium channels by the fragile X mental retardation protein. *Neuron* 72 630–642 10.1016/j.neuron.2011.09.033 22099464 PMC3433402

[B81] Lee H. Lin M. C. Kornblum H. I. Papazian D. M. Nelson S. F. (2014). Exome sequencing identifies de novo gain of function missense mutation in KCND2 in identical twins with autism and seizures that slows potassium channel inactivation. *Hum. Mol. Genet.* 23 3481–3489 10.1093/hmg/ddu056 24501278 PMC4049306

[B82] Lee U. S. Cui J. (2010). BK channel activation: structural and functional insights. *Trends Neurosci.* 33 415–423 10.1016/j.tins.2010.06.004 20663573 PMC2929326

[B83] Lopatin A. N. Makhina E. N. Nichols C. G. (1994). Potassium channel block by cytoplasmic polyamines as the mechanism of intrinsic rectification. *Nature* 372 366–369 10.1038/372366a0 7969496

[B84] Lu Z. Mac Kinnon R. (1994). Electrostatic tuning of Mg2+ affinity in an inward-rectifier K^+^ channel. *Nature* 371 243–246 10.1038/371243a0 7915826

[B85] Maljevic S. Wuttke T. V. Lerche H. (2008). Nervous system KV7 disorders: breakdown of a subthreshold brake. *J. Physiol.* 586 1791–801 10.1113/jphysiol.2008.150656 18238816 PMC2375730

[B86] Marrion N. V. Tavalin S. J. (1998). Selective activation of Ca2^+^- activated K^+^ channels by co-localized Ca2^+^ channels in hippocampal neurons. *Nature* 395 900–905 10.1038/27674 9804423

[B87] Matsuda H. Saigusa A. Irisawa H. (1987). Ohmic conductance through the inwardly rectifying K^+^ channel and blocking by internal Mg2^+^. *Nature* 325 156–159 10.1038/325156a0 2433601

[B88] Matsuo M. Maeda T. Sasaki K. Ishii K. Hamasaki Y. (2010). Frequent association of autism spectrum disorder in patients with childhood onset epilepsy. *Brain Dev.* 32 759–763 10.1016/j.braindev.2010.05.005 20542395

[B89] Michalon A. Sidorov M. Ballard T. M. Ozmen L. Spooren W. Wettstein J. G. (2012). Chronic pharmacological mGlu5 inhibition corrects Fragile X in adult mice. *Neuron* 74 49–56 10.1016/j.neuron.2012.03.009 22500629 PMC8822597

[B90] Mikhailov A. Choufani S. Skaug J. Kolozsvari D. Marshall C. Scherer S. W. (2008). Chromosomal translocation t(5;7)(q14;q31) and missense mutations implicate the voltage-gated potassium channel Kv4.2 gene, KCND2, on 7q31 in autism. *Paper Presented at the Annual Meeting of the American Society of Human Genetics* Philadelphia, PA.

[B91] Mongiat L. A. Espòsito M. S. Lombardi G. Schinder A. F. (2009). Reliable activation of immature neurons in the adult hippocampus. *PLoS ONE* 4:e5320 10.1371/journal.pone.0005320 PMC2670498 19399173

[B92] Mouridsen S. E. Rich B. Isager T. (2011). A longitudinal study of epilepsy and other central nervous system diseases in individuals with and without a history of infantile autism. *Brain Dev.* 33 361–366 10.1016/j.braindev.2010.07.002 20655678

[B93] Muddashetty R. Kelić S. Gross C. Xu M. Bassell G. (2007). Dysregulated metabotropic glutamate receptor-dependent translation of AMPA receptor and postsynaptic density-95 mRNAs at synapses in a mouse model of fragile X syndrome. *J. Neurosci.* 27 5338–5348 10.1523/JNEUROSCI.0937-07.2007 17507556 PMC6672337

[B94] Nerbonne J. M. Gerber B. R. Norris A. Burkhalter A. (2008). Electrical remodelling maintains firing properties in cortical pyramidal neurons lacking KCND2-encoded A-type K^+^ currents. *J. Physiol.* 586 1565–1579 10.1113/jphysiol.2007.146597 18187474 PMC2375705

[B95] Neusch C. Rozengurt N. Jacobs R. E. Lester H. A. Kofuji P. (2001). Kir4.1 potassium channel subunit is crucial for oligodendrocyte development and in vivo myelination. *J. Neurosci.* 21 5429–5438. 11466414 10.1523/JNEUROSCI.21-15-05429.2001 PMC6762664

[B96] Okamoto N. Hatsukawa Y. Shimojima K. Yamamoto T. (2011). Submicroscopic deletion in 7q31 encompassing CADPS2 and TSPAN12 in a child with autism spectrum disorder and PHPV. *Am. J. Med. Genet. A.* 155A 1568–1573 10.1002/ajmg.a.34028 21626674

[B97] Orkand R. K. Nicholls J. G. Kuﬄer S. W. (1966). Effect of nerve impulses on the membrane potential of glial cells in the central nervous system of amphibia. *J. Neurophysiol.* 29 788–806. 5966435 10.1152/jn.1966.29.4.788

[B98] O’Roak B. J. Deriziotis P. Lee C. Vives L. Schwartz J. J. Girirajan S. (2011). Exome sequencing in sporadic autism spectrum disorders identifies severe de novo mutations. *Nat. Genet.* 43 585–589 10.1038/ng.835 21572417 PMC3115696

[B99] Pessia M. (2004). “Ion channels and electrical activity,” in *Molecular Biology of the Neuron* 2nd Edn eds Davies W. R. Morris B. J. (Oxford: Oxford University Press) 103–137 10.1093/acprof:oso/9780198509981.003.0005

[B100] Pessia M. Imbrici P. D’Adamo M. C. Salvatore L. Tucker S. J. (2001). Differential pH-sensitivity of Kir4.1and Kir4.2 and modulation by heteropolymerisation with Kir5.1. *J. Physiol.* 532 359–367 10.1111/j.1469-7793.2001.0359f.x 11306656 PMC2278540

[B101] Pessia M. Tucker S. J. Lee K. Bond C. T. Adelman J. P. (1996). Subunit positional effects revealed by novel heteromeric inwardly rectifying K^+^ channels. *EMBO J.* 15 2980–2987. 8670799 PMC450239

[B102] Pieretti M. Zhang F. P. Fu Y. H. Warren S. T. Oostra B. A. Caskey C. T. (1991). Absence of expression of the FMR-1 gene in fragile X syndrome. *Cell* 66 817–822 10.1016/0092-8674(91)90125-I 1878973

[B103] Reichold M. Zdebik A. A. Lieberer E. Rapedius M. Schmidt K. Bandulik S. (2010). KCNJ10 gene mutations causing EAST syndrome (epilepsy, ataxia, sensorineural deafness, and tubulopathy) disrupt channel function. *Proc. Natl. Acad. Sci. U.S.A.* 107 14490–14495 10.1073/pnas.1003072107 20651251 PMC2922599

[B104] Rinehart N. McGinley J. (2010). Is motor dysfunction core to autism spectrum disorder? *Dev. Med. Child Neurol.* 52 697 10.1111/j.1469-8749.2010.03631.x 20345958

[B105] Rinehart N. J. Tonge B. J. Iansek R. McGinley J. Brereton A. V. Enticott P. G. (2006). Gait function in newly diagnosed children with autism: cerebellar and basal ganglia related motor disorder. *Dev. Med. Child Neurol.* 48 819–824 10.1017/S0012162206001769 16978461

[B106] Robitaille R. Charlton M. P. (1992). Presynaptic calcium signals and transmitter release are modulated by calcium-activated potassium channels. *J. Neurosci.* 12 297–305. 1370323 10.1523/JNEUROSCI.12-01-00297.1992 PMC6575681

[B107] Robitaille R. Garcia M. L. Kaczorowski G. J. Charlton M. P. (1993). Functional colocalization of calcium and calcium-gated potassium channels in control of transmitter release. *Neuron* 11 645–655 10.1016/0896-6273(93)90076-4 7691106

[B108] Safiulina V. F. Zacchi P. Taglialatela M. Yaari Y. Cherubini E. (2008). Low expression of Kv7/M channels facilitates intrinsic and network bursting in the developing rat hippocampus. *J. Physiol.* 586 5437–5453 10.1113/jphysiol.2008.156257 18801845 PMC2655386

[B109] Sanguinetti M. C. Tristani-Firouzi M. (2000). “Delayed and inward rectifier potassium channels,” in *Cardiac Electrophysiology From Cell to Bedside* 3rd Edn eds Zipes D. P. Jalife J. (Philadelphia, PA: Elsevier/W.B. Saunders) 79–85.

[B110] Santoro M. R. Bray S. M. Warren S. T. (2012). Molecular mechanisms of Fragile X syndrome: a twenty-year perspective. *Annu. Rev. Pathol.* 7 219–245 10.1146/annurev-pathol-011811-132457 22017584

[B111] Sausbier M. Hu H. Arntz C. Feil S. Kamm S. Adelsberger H. (2004). Cerebellar ataxia and Purkinje cell dysfunction caused by Ca2^+^-activated K^+^ channel deficiency. *Proc. Natl. Acad. Sci. U.S.A.* 101 9474–9478 10.1073/pnas.0401702101 15194823 PMC439001

[B112] Schaaf C. P. Zoghbi H. Y. (2011). Solving the autism puzzle a few pieces at a time. *Neuron* 70 806–808 10.1016/j.neuron.2011.05.025 21658575

[B113] Scherer S. W. Cheung J. MacDonald J. R. Osborne L. R. Nakabayashi K. Herbrick J. A. (2003). Human chromosome 7: DNA sequence and biology. *Science* 300 767–772 10.1126/science.1083423 12690205 PMC2882961

[B114] Schmunk G. Gargus J. J. (2013). Channelopathy pathogenesis in autism spectrum disorders. *Front. Genet.* 4:222 10.3389/fgene.2013.00222 PMC3817418 24204377

[B115] Scholl U. I. Choi M. Liu T. Ramaekers V. T. Häusler M. G. Grimmer J. (2009). Seizures sensorineural deafness ataxia mental retardation and electrolyte imbalance (SeSAME syndrome) caused by mutations in KCNJ10. *Proc. Natl. Acad. Sci. U.S.A.* 106 5842–5847 10.1073/pnas.0901749106 19289823 PMC2656559

[B116] Schroeder B. C. Hechenberger M. Weinreich F. Kubisch C. Jentsch T. J. (2000). KCNQ5, a novel potassium channel broadly expressed in brain, mediates M-type currents. *J. Biol. Chem.* 275 24089–24095 10.1074/jbc.M003245200 10816588

[B117] Seifert G. Hüttmann K. Binder D. K. Hartmann C. Wyczynski A. Neusch C. (2009). Analysis of astroglial K^+^ channel expression in the developing hippocampus reveals a predominant role of the Kir4.1 subunit. *J. Neurosci.* 29 7474–7488 10.1523/JNEUROSCI.3790-08.2009 19515915 PMC6665420

[B118] Shah M. M. Migliore M. Valencia I. Cooper E. C. Brown D. A. (2008). Functional significance of axonal Kv7 channels in hippocampal pyramidal neurons. *Proc. Natl. Acad. Sci. U.S.A.* 105 7869–7874 10.1073/pnas.0802805105 18515424 PMC2408483

[B119] Shao L. R. Halvorsrud R. Borg-Graham L. Storm J. F. (1999). The role of BK-type Ca2^+^-dependent K^+^ channels in spike broadening during repetitive firing in rat hippocampal pyramidal cells. *J. Physiol.* 521 135–146 10.1111/j.1469-7793.1999.00135.x 10562340 PMC2269638

[B120] Sibille J. Pannasch U. Rouach N. (2014). Astroglial potassium clearance contributes to short-term plasticity of synaptically evoked currents at the tripartite synapse. *J. Physiol.* 592 87–102 10.1113/jphysiol.2013.261735 24081156 PMC3903353

[B121] Sicca F. Imbrici P. D’Adamo M. C. Moro F. Bonatti F. Brovedani P. (2011). Autism with seizures and intellectual disability: possible causative role of gain-of-function of the inwardly-rectifying K^+^ channel Kir4.1. *Neurobiol. Dis.* 43 239–247 10.1016/j.nbd.2011.03.016 21458570

[B122] Singh B. Ogiwara I. Kaneda M. Tokonami N. Mazaki E. Baba K. (2006). A Kv4.2 truncation mutation in a patient with temporal lobe epilepsy. *Neurobiol. Dis.* 24 245–253 10.1016/j.nbd.2006.07.001 16934482

[B123] Singh N. A. Otto J. F. Dahle E. J. Pappas C. Leslie J. D. Vilaythong A. (2008). Mouse models of human KCNQ2 and KCNQ3 mutations for benign familial neonatal convulsions show seizures and neuronal plasticity without synaptic reorganization. *J. Physiol.* 586 3405–3423 10.1113/jphysiol.2008.154971 18483067 PMC2538806

[B124] Singh N. A. Westenskow P. Charlier C. Pappas C. Leslie J. Dillon J. (2003). KCNQ2 and KCNQ3 potassium channel genes in benign familial neonatal convulsions: expansion of the functional and mutation spectrum. *Brain* 126 2726–2737 10.1093/brain/awg286 14534157

[B125] Siomi H. Siomi M. C. Nussbaum R. L. Dreyfuss G. (1993). The protein product of thefragile X gene, FMR1, has characteristics of an RNA-binding protein. *Cell* 74 291–298 10.1016/0092-8674(93)90420-U 7688265

[B126] Stanfield P. R. Davies N. W. Shelton P. A. Khan I. A. Brammar W. J. Standen N. B. (1994). The intrinsic gating of inward rectifier K^+^ channels expressed from the murine IRK1gene depends on voltage, K^+^, and Mg2^+^. *J. Physiol.* 475 1–7 10.1113/jphysiol.1994.sp020044 8189383 PMC1160350

[B127] Steinhäuser C. Grunnet M. Carmignoto G. (2015). Crucial role of astrocytes in temporal lobe epilepsy. *Neuroscience* 10.1016/j.neuroscience.2014.12.047 [Epub ahead of print]. 25592426

[B128] Steinlein O. K. Conrad C. Weidner B. (2007). Benign familial neonatal convulsions: always benign? *Epilepsy Res.* 73 245–249 10.1016/j.eplepsyres.2006.10.010 17129708

[B129] Steinlein O. K. Mulley J. C. Propping P. Wallace R. H. Phillips H. A. Sutherland G. R. (1995). A missense mutation in the neuronal nicotinic acetylcholine receptor alpha 4 subunit is associated with autosomal dominant nocturnal frontal lobe epilepsy. *Nat. Genet.* 11 201–203 10.1038/ng1095-201 7550350

[B130] Storm J. F. (1987). Intracellular injection of a Ca2+ chelator inhibits spike repolarization in hippocampal neurons. *Brain Res.* 435 387–392 10.1016/0006-8993(87)91631-3 3123013

[B131] Takumi T. Ishii T. Horio Y. Morishige K. Takahashi N. Yamada M. (1995). A novel ATP-dependent inward rectifier potassium channel expressed predominantly in glial cells. *J. Biol. Chem.* 270 16339–16346 10.1074/jbc.270.27.16339 7608203

[B132] Tempel B. L. Papazian D. M. Schwarz T. L. Jan Y. N. Jan L. Y. (1987). Sequence of a probable potassium channel component encoded at Shaker locus of *Drosophila*. *Science* 237 770–775 10.1126/science.2441471 2441471

[B133] Todd P. Malter J. (2002). Fragile X mental retardation protein in plasticity and disease. *J. Neurosci. Res.* 70 623–630 10.1002/jnr.10453 12424729

[B134] Tovia E. Goldberg-Stern H. Ben Z. B. Heyman E. Watemberg N. Fattal- Valevski A. (2011). The prevalence of atypical presentations and comorbidities of benign childhood epilepsy with centrotemporal spikes. *Epilepsia* 52 1483–1488 10.1111/j.1528-1167.2011.03136.x 21692792

[B135] Tuchman R. Rapin I. (2002). Epilepsy in autism. *Lancet Neurol.* 1 352–358 10.1016/S1474-4422(02)00160-6 12849396

[B136] Tucker J. S. Imbrici P. Salvatore L. D’Adamo M. C. Pessia M. (2000). pH-dependence of the inwardly rectifying potassium channel Kir5.1and localisation in renal tubular epithelia. *J. Biol. Chem.* 275 16404–16407 10.1074/jbc.C000127200 10764726

[B137] Van Waelvelde H. Oostra A. Dewitte G. Van Den Broeck C. Jongmans M. J. (2010). Stability of motor problems in young children with or at risk of autism spectrum disorders, ADHD, and or developmental coordination disorder. *Dev. Med. Child Neurol.* 52 e174–e178 10.1111/j.1469-8749.2009.03606.x 20132135

[B138] Verheij C. Bakker C. E. de Graaff E. Keulemans J. Willemsen R. Verkerk A. J. (1993). Characterization and localization of the FMR-1 gene product associated with fragile X syndrome. *Nature* 363 722–724 10.1038/363722a0 8515814

[B139] Verheij J. B. de Munnik S. A. Dijkhuizen T. de Leeuw N. Olde Weghuis D. van den Hoek G. J. (2009). An 8.35 Mb overlapping interstitial deletion of 8q24 in two patients with coloboma, congenital heart defect, limb abnormalities, psychomotor retardation and convulsions. *Eur. J. Med. Genet.* 52 353–357 10.1016/j.ejmg.2009.05.006 19464398

[B140] Wang H. S. Pan Z. Shi W. Brown B. S. Wymore R. S. Cohen I. S. (1998). KCNQ2 and KCNQ3 potassium channel subunits: molecular correlates of the M-channel. *Science* 282 1890–1893 10.1126/science.282.5395.1890 9836639

[B141] Wang S. Patel S. P. Qu Y. Hua P. Strauss H. C. Morales M. J. (2002). Kinetic properties of Kv4.3 and their modulation by KChIP2b. *Biochem. Biophys. Res. Commun.* 295 223–229 10.1016/S0006-291X(02)00658-7 12150935

[B142] Wegiel J. Kuchna I. Nowicki K. Imaki H. Wegiel J. Ma S. Y. (2013). Contribution of olivofloccular circuitry developmental defects to atypical gaze in autism. *Brain Res.* 1512 106–122 10.1016/j.brainres.2013.03.037 23558308 PMC3967119

[B143] Wei A. D. Gutman G. A. Aldrich R. Chandy K. G. Grissmer S. Wulff H. (2005). International Union of Pharmacology LII Nomenclature and molecular relationships of calcium- activated potassium channels. *Pharmacol. Rev.* 57 463–472 10.1124/pr.57.4.9 16382103

[B144] Weiler I. J. Irwin S. A. Klintsova A. Y. Spencer C. M. Brazelton A. D. Miyashiro K. (1997). Fragile X mental retardation protein is translated near synapses in response to neurotransmitter activation. *Proc. Natl. Acad. Sci. U.S.A.* 94 5395–5400 10.1073/pnas.94.10.5395 9144248 PMC24689

[B145] Weiler I. Spangler C. Klintsova A. Grossman A. Kim S. Bertaina-Anglade V. (2004). Fragile X mental retardation protein is necessary for neurotransmitter activated protein translation at synapses. *Proc. Natl. Acad. Sci. U.S.A* 101 17504–17509 10.1073/pnas.0407533101 15548614 PMC536018

[B146] Weiss L. A. Escayg A. Kearney J. A. Trudeau M. MacDonald B. T. Mori M. (2003). Sodium channels SCN1A, SCN2A and SCN3A in familial autism. *Mol. Psychiatry* 8 186–194 10.1038/sj.mp.40x01241 12610651

[B147] Womack M. D. Hoang C. Khodakhah K. (2009). Large conductance calcium-activated potassium channels affect both spontaneous firing and intracellular calcium concentration in cerebellar Purkinje neurons. *Neuroscience* 162 989–1000 10.1016/j.neuroscience.2009.05.016 19446607 PMC2723190

[B148] Xia X. M. Fakler B. Rivard A. Wayman G. Johnson-Pais T. Keen J. E. (1998). Mechanism of calcium gating in small conductance calcium-activated potassium channels. *Nature* 395 503–507 10.1038/26758 9774106

[B149] Yu F. H. Mantegazza M. Westenbroek R. E. Robbins C. A. Kalume F. Burton K. A. (2006). Reduced sodium current in GABAergic interneurons in a mouse model of severe myoclonic epilepsy in infancy. *Nat. Neurosci.* 9 1142–1149 10.1038/nn1754 16921370

[B150] Zalfa F. Giorgi M. Primerano B. Moro A. Di Penta A. Reis S. (2003). The fragile X syndrome protein FMRP associates with BC1 RNA and regulates the translation of specific mRNAs at synapses. *Cell* 112 317–327 10.1016/S0092-8674(03)00079-5 12581522

[B151] Zhao C. Wang L. Netoff T. Yuan L. L. (2011). Dendritic mechanisms controlling the threshold and timing requirement of synaptic plasticity. *Hippocampus* 21 288–297 10.1002/hipo.20748 20087888

[B152] Zhu X. R. Wulf A. Schwarz M. Isbrandt D. Pongs O. (1999). Characterization of human Kv4.2 mediating a rapidly-inactivating transient voltage-sensitive K^+^ current. *Receptors Channels* 6 387–400. 10551270

